# Medical and Physical Intelligence

**Published:** 1801-12

**Authors:** 


### MEDICAL AND PHYSICAL INTELLIGENCE.

### [ FOREIGN AND DOMESTIC. ]

### *On the Nature and Generation of the earthy Particles in different Kinds of indigenous Corn.*

The Royal Pruſſian Academy of Sciences at Berlin, a few months ago, propoſed the following prize-queſtion:

" Of what kind are the terreous particles that are found by a " chemical analyſis in the different ſorts of indigenous corn? Do " they enter into them as ſuch, or are they produced in them by " the *vis vitalis* and the action of the organs of vegetation?"

The Academy has ſince publiſhed two papers, that were delivered upon the ſubject; one of which, by Mr. Schrader, Apothecary at Berlin, deſerves our notice. — The firſt queſtion he has endeavoured to anſwer by ſeveral chemical analyſis made for the purpoſe. For decompoſing the different grains into their earthy particles, *combuſtion* and *calcination*, or *incineration*, was thought the moſt proper and convenient method. The aſhes obtained in this way were very pure, and without any heterogeneous mixture; and being afterwards farther treated with chemical *reagency*, in order to extract their earthy particles, the following reſults were found:

32 ounces of oats afforded:

| | | |
|---|---|---|
| Siliceous earth — — — | $144\frac{2}{10}$ | grains. |
| Carbonat of lime — — — | $33\frac{15}{20}$ | |
| Carbonat of magneſia — — | $33\frac{9}{10}$ | |
| Aluminous earth — — — | $4\frac{5}{10}$ | |
| Mangaueſe in the ſtate of oxyd — | $4\frac{5}{10}$ | |
| Iron in the ſtate of Pruſſian blue — | $4\frac{5}{10}$ | |

32 ounces of rye afforded:

| | | |
|---|---|---|
| Siliceous earth — — — | $15\frac{6}{10}$ | grains. |
| Carbonat of lime — — — | $13\frac{4}{10}$ | |
| Carbonat of magneſia — — | $14\frac{2}{10}$ | |
| Aluminous earth — — — | $1\frac{4}{10}$ | |
| Manganeſe in the ſtate of oxyd — | $3\frac{2}{10}$ | |
| Iron in the ſtate of Pruſſian blue — | $0\frac{9}{10}$ | |

32 ounces of barley afforded:

| | | |
|---|---|---|
| Siliceous earth — — — | $66\frac{7}{10}$ | grains. |
| Carbonat of lime — — — | $24\frac{8}{10}$ | |
| Carbonat of magneſia — — | $25\frac{3}{10}$ | |
| Manganeſe in the ſtate of oxyd — | $6\frac{7}{10}$ | |
| Iron in the ſtate of Pruſſian blue — | $3\frac{8}{10}$ | |

32 ounces

32 ounces of wheat afforded:

| | | | | | |
|---|---|---|---|---|---|
| Siliceous earth | — | — | — | $13\frac{2}{10}$ | grains. |
| Carbonat of lime | — | — | — | $12\frac{6}{10}$ | |
| Carbonat of magnefia | | — | — | $13\frac{4}{10}$ | |
| Oxyd of manganefe | — | — | — | 5 | |
| Aluminous earth | — | — | — | $0\frac{6}{10}$ | |
| Iron in the ftate of Pruffian blue | | | — | $2\frac{5}{10}$ | |

32 ounces of rye ftraw afforded:

| | | | | | |
|---|---|---|---|---|---|
| Siliceous earth | — | — | — | 152 | grains. |
| Carbonat of lime | — | — | — | $46\frac{2}{10}$ | |
| Carbonat of magnefia | | — | — | $28\frac{2}{10}$ | |
| Aluminous earth | — | — | — | $3\frac{2}{10}$ | |
| Oxyd of manganefe | — | — | — | $6\frac{8}{10}$ | |
| Iron in the ftate of Pruffian blue | | | — | $2\frac{4}{10}$ | |

The fecond part of the queftion is fubject to more difficulties. Mr. Schrader thought to afcertain it beft by letting thofe vegetables grow in media, which were quite deprived of all earthy particles; and by inquiring afterwards whether they contained earths, or by comparing their conftituents with the conftituents of thofe that had been growing in earth. For this purpofe, therefore, Mr. Schrader employed, after having tried feveral other fubftances, the fublimed fulphur, into which, after being wafhed with diftilled water, he fowed rye, becaufe this feemed to thrive beft of all, and a little barley. The veffels which he ufed were of glafs or porcelaine. He expofed them to free air, and to the fun in a garden, where no duft could come to them; and, in order to fecure them ftill better from its accefs, the veffels were put under glafs frames, which were only a little opened. For watering them he ufed diftilled water, impregnated with carbonic acid. The feafon in which the experiment was made being already much advanced, he was obliged to let the plants continue to grow till near the end of autumn. Some of the plants had reached the height of 12 to 14 inches, and feveral had produced fmall ears, in which, however, the parts of fructification could be clearly diftinguifhed. There were in all about 28 plants of rye, and 12 of barley, which weighed together about 108 grains. After being reduced to cinder in a filver crucible, they were burned with faltpetre, in which procefs two grains of earthy particles were obtained. 108 grains of rye ftraw gave, when burned with faltpetre, 2 grains of earth; and 28 corns of rye, and 12 of barley, $\frac{5}{10}$ grains of earth. By thefe experiments it was therefore afcertained, that 40 plants produced in fulphur, contained an equal quantity of earthy particles with thofe which had grown in earth. Mr. Schrader enquired now, by another analyfis, as accurately as it was poffible to do with fuch a fmall quantity, if the earthy particles obtained from thofe plants were of the fame kind as the plants which had thriven in common earth, and this was confirmed by his experiments. From thefe facts it may be juftly concluded, that the earthy and metallic particles contained in different fpecies of corn, do not enter into them as fuch, but are generated in them by the organic action of vegetation.

*Change of metallic Bodies by hot Lava.* When the town *Terre del Greco*, near Naples, had the misfortune of being deftroyed and covered with lava, on clearing the place again, feveral metallic inftruments were found to have undergone a curious change. Prof. Klaproth, of Berlin, had an opportunity of receiving feveral productions of that kind, and, amongft others, the fragment of a bell, in which the two metals that compofe the bell metal, were entirely feparated, the tin having difappeared, while the copper had the appearance of red copper ore, covered with a ftratum of pure copper; the lava furrounding it was intermixed with dark grey fteel coloured cryftals, of the appearance of copper glofs ore and red copper ore. On the furface of another bell, a group of cryftals had formed itfelf, perfectly fimilar to folid copper glofs ore, in the fhape of quadrangular pointed columns. He alfo received the lower end of the barrel of a gun, together with the lock; the iron of which had entirely loft its metallic ftate, being changed into a kind of martial ethiops, whereby the fize of the whole had confiderably increafed, almoft to more than three times its former fize. When broke, it had a metallic glofs of a blackifh colour, partaking a little of red; the texture was coarfe and fomewhat fcaly. The fpecific weight was confiderably diminifhed, while the abfolute weight had become much greater, the former being only 4,848, whereas the fpecific weight of the pure metallic iron amounts to 7,783 = 7,800. In order to determine the increafe of the abfolute weight, or the quantity of admixed oxygen, 100 parts of iron were diffolved in muriatic acid, and afterwards precipitated. The precipitation being edulcorated and dried, was changed into martial ethiops by heating it with a few drops of olive oil, by which means it was found to have increafed by 25 per cent. which, confequently is the fame cafe with the above barrel. This augmentation of weight is undoubtedly produced by the oxygen communicated to the iron from the decompofed vapours.

---

*Citizen Vauquelin* has analyfed another foffil, remarkable for the confiderable quantity of arfenic acid which it contains, and therefore ftiled by Mr. *Karften*, *Pharmacolith*. It is found in the country of Turftenberg in fmall, white, hair-like prifmatic cryftals, generally occuring in clufters or heaps, and their fpecific weight is = 2,640. The chief particles of thefe curious cryftals is a combination of lime with arfenic acid, or an arfeniat of lime, mixed with fome adventitious particles, as cobalt and filiceous earth, in the following proportion:

| | |
|---|---|
| Arfenic acid — — | 46, 50 |
| Lime — — — | 23, |
| Oxyd of cobalt — — | 0, 50 |
| Siliceous earth — — | 6, |
| Water — — — | 22, 46 |
| | 98, 50 |

The

The proportion of the effential particles after the fubftraction of the adventitious ones is,

| | | |
|---|---|---|
| Arfenic acid | — — | 50, 54 |
| Lime | — — — | 25, |
| Water | — — — | 24, 46 |
| | | 100 |

---

The great ufe of blifters applied on the fhaved head in fome cafes of delirium, is fufficiently known to practitioners, but a remarkable inftance of that kind is recorded in *Hufeland's* ufeful Journal for Practical Medicine, Vol. XI. No. IV. The patient perfectly recovered his fenfes the next morning, after a blifter had been put on the fhaved head the night before. The excoriated places were kept open with unguent. bafilic. and pulvis cantharid. for ten days; and when the patient feemed perfectly reafonable, the wound was dreffed with ceratum Saturni, in order to heal it up, which however, having hardly been effected, the patient was again feized with delirious fits, which increafed to fuch a degree, that recourfe was had to another blifter, which being kept in fuppuration for three weeks, the patient was entirely reftored to health. Befides the blifters, proper internal remedies had been employed. The editor of the above Journal relates, in addition to this, a cafe of apoplexy, in which the application of an actual cautery proved moft efficacious. "A lady having been taken with an apopletic fit, lay in a foporous ftate, againft which all poffible remedies had been employed in vain by the attending phyfician, when a friend of the patient recommended to apply paper burnt to tinder on the fhaved head. This had hardly been done, when the patient arofe with vehemence, tore off the cap that had caught fire, and cried with a ftrong voice, "Will you burn me!" Againft the firft intention, the cap had been fet on fire by fome glowing points in the tinder that were overlooked; and though the fkin on the head of the patient was confiderably burnt, the patient had the advantage of being perfectly recovered.

---

Amongft the antagonifts that have arofe againft the Vaccine Inoculation in Germany, none feems to excite fo much the attention of the public, as Dr. *Marcus Herz* of Berlin, one of the moft refpectable phyficians of Germany, and juftly celebrated by feveral of his publications. This gentleman has lately publifhed a memoir in *Hufeland's* Medical Journal, Vol. XII. No. I. which is alfo feparately to be had, entitled, "*Dr. Marcus Herz to Dr. Dohmeyer,* phyfician to his Royal Highnefs Prince Auguftus of England, *on the Brutal Inoculation, and its comparifon with the Human Inoculation,* (in German) with the following motto prefixed to it."

Homo fum, non humana a me aliena puto.

The ingenious author of this treatife has exhaufted every argument that may theoretically be fuggefted againft introducing a brutal poifon into the human conftitution, and reafoned on this fubject with fuch acutenefs and ingenuity, as might be expected from fo philofophilosophical a physician. The evidence of facts, however, on which the great use of Vaccine Inoculation is grounded, is not so easily to be subverted by the most subtle reasoning; and though it may contribute to draw the attention of physicians on several subjects attending that great discovery, and fix several points in view hitherto neglected, yet it is by no means calculated to make us desist from farther pursuiug and acertaining an object of such extensive advantage for the benefit of the community, and which seems, by the numerous experiments it has undergone, to have reached the summit of certainty and truth. As we purpose to lay before our readers, in a future Number of this Journal, a full account of all publications that have appeared on the Vaccine Inocnlation in Germany, we forbear at present to state any thing farther respecting this subject, and only beg leave to mention, that several pamphlets have been purposely written, in order to oppose the more subtle than true argumentation of *Dr. Herz*.

